# Monitor-based exoscopic 3D4k neurosurgical interventions: a two-phase prospective-randomized clinical evaluation of a novel hybrid device

**DOI:** 10.1007/s00701-020-04361-2

**Published:** 2020-05-19

**Authors:** Anna L. Roethe, Philipp Landgraf, Torsten Schröder, Martin Misch, Peter Vajkoczy, Thomas Picht

**Affiliations:** 1 Department of Neurosurgery, Charité-Universitätsmedizin Berlin, Charitéplatz 1, 10117 Berlin, Germany; 2 Interdisciplinary Laboratory Image Knowledge Gestaltung, Humboldt-Universität zu Berlin, Berlin, Germany; 3 Department of Anesthesiology and Operative Intensive Care Medicine, Charité-Universitätsmedizin Berlin, Berlin, Germany

**Keywords:** Brain tumor, Digital innovation, Exoscope, Intraoperative visualization, Technology evaluation

## Abstract

**Background:**

Promoting a disruptive innovation in microsurgery, exoscopes promise alleviation of physical strain and improved image quality through digital visualization during microneurosurgical interventions. This study investigates the impact of a novel 3D4k hybrid exoscope (i.e., combining digital and optical visualization) on surgical performance and team workflow in preclinical and clinical neurosurgical settings.

**Methods:**

A pre-clinical workshop setting has been developed to assess usability and implementability through skill-based scenarios (neurosurgical participants *n* = 12). An intraoperative exploration in head and spine surgery (*n* = 9) and a randomized clinical study comparing ocular and monitor mode in supratentorial brain tumor cases (*n* = 20) followed within 12 months. Setup, procedure, case characteristics, surgical performance, and user experience have been analyzed for both ocular group (OG) and monitor group (MG).

**Results:**

Brain tumor cases using frontal, frontoparietal, or temporal approaches have been identified as favorable use cases for introducing exoscopic neurosurgery. Mean monitor distance and angle were 180 cm and 10°. Surgical ergonomics when sitting improved significantly in MG compared with OG (*P* = .03). Hand-eye coordination required familiarization in MG. Preclinical data showed a positive correlation between lateral camera inclination and impact on hand-eye coordination (*r*_s_ = 0.756, *P* = .01). There was no significant added surgical time in MG. Image quality in current generation 3D4k monitors has been rated inferior to optic visualization yet awaits updates.

**Conclusions:**

The hybrid exoscopic device can be integrated into established neurosurgical workflows. Currently, exoscopic interventions seem most suited for cranial tumor surgery in lesions that are not deep-seated. Ergonomics improve in monitor mode compared to conventional microsurgery.

**Electronic supplementary material:**

The online version of this article (10.1007/s00701-020-04361-2) contains supplementary material, which is available to authorized users.

## Introduction

Microsurgery is being facing a new trend: the convergence of endoscopic and microscopic visualization principles resulting in lighter and more versatile surgical exoscopes. From the clinical perspective, this device evolution is designed to address current constraints in surgical access and ergonomics, digitally enhanced visualization and information sharing in the OR, all of which can have high impact on surgical interventions of varying complexity. Exoscope technology is trying to overcome previously experienced limitations in endoscopy and surgical microscopy [25, 26] by merging these two major intraoperative visualization techniques [4, 22] and seeking to establish new quality standards [4]. An exoscope is an extracorporeal scope [13] distanced from the surgical site, producing high-quality video images with a wide field of view [19]. Its components comprise a tubular telescope or microscope, a camera unit, a light source, a monitor, and a control unit. For many exoscopes directly base on the principles of endoscopy, they reproduce its inherent dissociation of working and viewing direction. In return, these machines promise to facilitate improvements in OR team visualization and surgical ergonomics [29]. Preclinical and clinical data is available for six exoscopic devices by different manufacturers (see Table 1), all of which benefit from recent technology improvements. However, disruptive effects on surgical workflow and intraoperative visualization requirements remain topics yet to be addressed more thoroughly. The introduction of the first digital hybrid visualization device combining exoscopic and microscopic features allowed for direct comparison of conventional and exoscopic surgery. To assist its clinical investigation, we set up a mixed method-study design focusing on identification of eligible cases, assessment of usability, analysis of device impact on surgical performance and team workflows, and exploration of visual quality standards in microneurosurgery.

**Table 1:** Preclinical and clinical investigation of exoscopic devices in neurosurgery 2008–2018 (*n* = 23)

Year	Paper	Type of study	Exoscope	Device characteristics	Type of surgery (*n*)	Evaluation of exoscope
2018	Takahashi et al. [29]	Clinical	ORBEYE (Olympus, Tokyo, Japan)	3D4k, 55″ monitor, 3D glasses, working distance 22-55 cm, FCP or manual control	Cranial (tumor, neurovascular) (*n* = 14)	(+) Setup, size, ergonomics, combination with navigation, HQ visuals for entire team (−) Integration of surgical assistant (rotated view)
2018	Kwan et al. [17]	Clinical	ORBEYE (Olympus, Tokyo, Japan)	3D4k, 55″ monitor, 3D glasses, working distance 22-55 cm, FCP or manual control	Spinal (mainly laminectomies) (*n* = 10)	(+) Ergonomics, size, weight, maneuverability, single hand manipulation, depth of field, immersion, HQ visuals for entire team, education (−) Scope adjustments, controls, case length, learning curve, monitor positioning when additional equipment
2018	Belykh et al. [4]	Preclinical (laboratory study)	ROVOT-M/ BRIGHTMATTER SERVO (Synaptive Medical, Toronto, Ontario, Canada) / KINEVO 900 (Carl Zeiss AG, Oberkochen, Germany)	2DHD, 55″ monitor, working distance n/A, manual control + FCP / 3D4k, 55″ monitor, working distance 20–62,5 cm, FCP or manual control	Neurovascular techniques	KINEVO 900 (+) 3D Depth perception (−) Depth perception and details at high magnification, physiologic eye accommodation, field of view ROVOT-M (+) Size (−) Stereopsis both (+) High resolution, ergonomics, education (−) 3D glasses, slight delay, learning curve
2018	Bakhsheshian et al. [1]	Clinical	VITOM (KARL STORZ-Endoscopy America, Inc., El Segundo, California, USA) / ROVOT-M/ BRIGHTMATTER SERVO (Synaptive Medical, Toronto, Ontario, Canada)	2DHD, 0- or 90-degree, 26″ monitor, working distance 25-75 cm, manual control/2DHD, 55″ monitor, working distance n/A, manual control + FCP	Cranial (subcortical brain metastases) (*n* = 25)	(+) Depth of field, ergonomics (−) None
2018	Gassie et al. [9]	Clinical	VITOM (Karl Storz Endoscopy America, Inc., El Segundo, California, USA)	2DHD, 0- or 90-degree, 26″ monitor, working distance 25-75 cm, manual control	Cranial (subcortical tumors) (*n* = 50)	(+) Ergonomics, wider field of view (−) Visualization angle, depth interpretation, micro adjustments of working field
2018	Beez et al. [2]	Clinical	VITOM 3D (Karl Storz GmbH, Tuttlingen, Germany)	3D4k, 32″ monitor, 3D glasses, working distance 20-50 cm, manual control (Joystick)	Cranial and spinal, pediatric neurosurgery (lesion, tumor, myelomeningocele) (*n* = 3)	(+) Ergonomics, accessibility of the surgical field, immersion, size, cost savings, HQ visuals for entire team, education (−) Deep lesions, illumination, lateral inclination of scope, adjustment controls, user-friendliness, assistant integration
2018	Klinger et al. [15]	Clinical	ROVOT-M/ BRIGHTMATTER SERVO (Synaptive Medical, Toronto, Ontario, Canada)	2DHD, 55″ monitor, working distance n/A, manual control + FCP	Cranial (aneurysm) (*n* = 1)	(+) Ergonomics, size, maneuverability, unobstructed surgical field, visualization of difficult angles/ trajectories, HQ visuals for entire team, ease of use, magnification, education (−) Steep learning curve, stereopsis, depth perception
2018	Sack et al. [26]	Preclinical (cadaver study)	ORBEYE (Olympus, Tokyo, Japan)	3D4k, 55″ monitor, 3D glasses, working distance 22-55 cm, FCP or manual control	Cranial (exposure and dissection) (*n* = 7)	(+) Optics, depth of field, ergonomics, size, single hand manipulation, no balancing required, maneuverability, immersion, HQ visuals for entire team, education, cost savings (−) Learning curve, monitor setup, blocking of line of sight by device
2017	Rossini et al. [25]	Clinical	VITOM 3D (Karl Storz GmbH, Tuttlingen, Germany)	3D4k, 32″ monitor, 3D glasses, working distance 20-50 cm, manual control (Joystick)	Cranial (meningioma) (*n* = 1)	(+) Ergonomics, size, maneuverability, weight, depth of field, 3D vision, color filter, magnification, microscopic-macroscopic switch, HQ visuals for entire team, education, cost savings (−) Repositioning, refocusing, magnification, second screen needed, controls
2017	Oertel/Burkhardt [23]	Clinical	VITOM 3D (Karl Storz GmbH, Tuttlingen, Germany)	3D4k, 32″ monitor, 3D glasses, working distance 20-50 cm, manual control (Joystick)	Cranial and spinal (tumor, fusion, decompression, discectomy) (*n* = 16)	(+) Ergonomics, size, maneuverability, depth of field, cost savings, education (−) Repositioning, identification of bleeding vessels
2017	Nishiyama [22]	Review	VITOM (Karl Storz Endoscopy America, Inc., El Segundo, California, USA)	2DHD, 0- or 90-degree, 26″ monitor, working distance 25-75 cm, manual control	Spinal (lipoma) (*n* = 1)	(+) Wide field of view, deep focus, less repositioning (−) Magnification, digital zoom, stereopsis
2017	Moisi et al. [21]	Preclinical (cadaver study)	ROVOT-M/ BRIGHTMATTER SERVO (Synaptive Medical, Toronto, Ontario, Canada)	2DHD, 55″ monitor, working distance n/A, manual control + FCP	Spinal (unilateral, single-level laminotomies) (*n* = 22)	(+)Ergonomics, depth of field, maneuverability, education, no added time (−) Stereopsis
2017	Jackson et al. [13]	Clinical	VITOM (Karl Storz Endoscopy America, Inc., El Segundo, California, USA)	2DHD, 0- or 90-degree, 26″ monitor, working distance 25-75 cm, manual control	Cranial (deep seated lesions with biopsy) (*n* = 11)	(+) Bimanual manipulation, flexibility, higher magnification (−) None
2017	Krishnan et al. [16]	Clinical	VITOM (Karl Storz Endoscopy America, Inc., El Segundo, California, USA)	2DHD, 0- or 90-degree, 26″ monitor, working distance 25-75 cm, manual control	Cranial and spinal (decompression, laminotomy, tumor, ICH) (*n* = 18)	(+) Ergonomics, size, magnification, illumination, HD images, angulation, depth of field (−) Adjustment and refocusing, added time, fluorescence filters, navigation, (stereopsis)
2017	Gonen et al. [11]	Clinical	ROVOT-M/BRIGHTMATTER SERVO (Synaptive Medical, Toronto, Ontario, Canada)	2DHD, 55″ monitor, working distance n/A, manual control + FCP	Cranial (tumors and ICH) (*n* = 200)	(+) No complications, robotic control (−) Learning curve, stereopsis
2016	Kassam et al. [14]	Preclinical (cadaver study), clinical	ROVOT-M/BRIGHTMATTER SERVO (Synaptive Medical, Toronto, Ontario, Canada)	2DHD, 55″ monitor, working distance n/A, manual control + FCP	Cranial (aneurysms) (*n* = 2 + 6)	(+) Larger immersive volume of surgical anatomy, preset positions, hand-free control (−) None
2014	Piquer et al. [24]	Clinical	HDXO-SCOPE (Karl Storz Endoscopy, Tuttlingen, Germany)	2DHD, 23″ monitor, working distance 20 cm, manual control	Cranial (tumors) (*n* = 38)	(+) Ergonomics, reduction of fatigue, fluorescence, wide field, HQ visuals for entire team, weight, cost savings (−) None
2014	Birch et al. [6]	Clinical	VITOM (Karl Storz Endoscopy, Tuttlingen, Germany)	2DHD, 90-degree, 23″ monitor, working distance 25-75 cm, manual control	Cranial (pineal lesions) (*n* = 5)	(+) Depth of field, ergonomics, HQ visuals for entire team, no added time (−) Stereopsis, assistant integration
2014	Belloch et al. [3]	Clinical	HDXO-SCOPE (Karl Storz Endoscopy, Tuttlingen, Germany)	2DHD, 23″ monitor, working distance 20 cm, manual control	Cranial (HGG) (*n* = 23)	(+) Cost savings, agility, weight, handling, ergonomics, microscopic-macroscopic switch, HQ visuals for entire team, sterilizable (−) None
2012	Shirzadi et al. [27]	Clinical	VITOM (Karl Storz Endoscopy, Tuttlingen, Germany)	2DHD, 0-degree, 23″ monitor, working distance 25-75 cm, manual control	Spinal (decompressions and interbody fusions) (*n* = 48)	(+) Optics, ergonomics, size, weight, versatility, interoperability, depth of field, no added time, HQ visuals for entire team, education, sterilizable (−) Stereopsis, repositioning
2012	Mamelak et al. [20]	Clinical	VITOM (Karl Storz Endoscopy, Tuttlingen, Germany)	2DHD, 23″ monitor, working distance 25-60 cm, manual control	Cranial (pineal lesion) (*n* = 1)	(+) Ergonomics, depth of field (−) Stereopsis, repositioning, focusing
2010	Mamelak et al. [19]	Clinical	HDXO-SCOPE (Karl Storz Endoscopy, Tuttlingen, Germany)	2DHD, 23″ monitor, working distance 20 cm, manual control	Cranial and spinal (mainly tumors) (*n* = 16)	(+) Ergonomics, magnification, HQ visuals for entire team, size, interoperability, cost savings, education (−) Stereopsis, repositioning, focusing, angulation, robotics
2008	Mamelak et al. [18]	Preclinical (animal model)	HDXO-SCOPE (Karl Storz Endoscopy, Tuttlingen, Germany)	2DHD, 23″ monitor, working distance 20 cm, manual control	Cranial (brain dissection in live pig model) (*n* = 4)	(+) Ergonomics, magnification, HQ visuals for entire team, size, interoperability, cost, sterilizable (−) Stereopsis, light intensity, telescope arm control, focus and zoom

*FCP* foot control panel, *HGG* high grade glioma, *HQ* high quality, *ICH* intracerebral hemorrhage

## Methods and materials

### System description

The hybrid visualization system assessed in this study (KINEVO 900, Carl Zeiss Meditec AG, Oberkochen, Germany) can be utilized as a conventional (optical) surgical microscope or as an exoscope with digital visualization on an external 55″ 3D4k monitor plus an internal 24″ 3DHD system monitor (utilizing passive 3D glasses). System specifications include a working distance of 200-625 mm, focal length of 170 mm, maximum magnification in exoscope mode 11, × 6, XY robotic movement in 6 axes and 3D4k stereo video cameras (2 × 3-chip 4 K, 2160p). Robotics can be controlled via a wireless 10-button plus joystick foot control panel (FCP).

### Study design and randomization

**Fig. 1:**
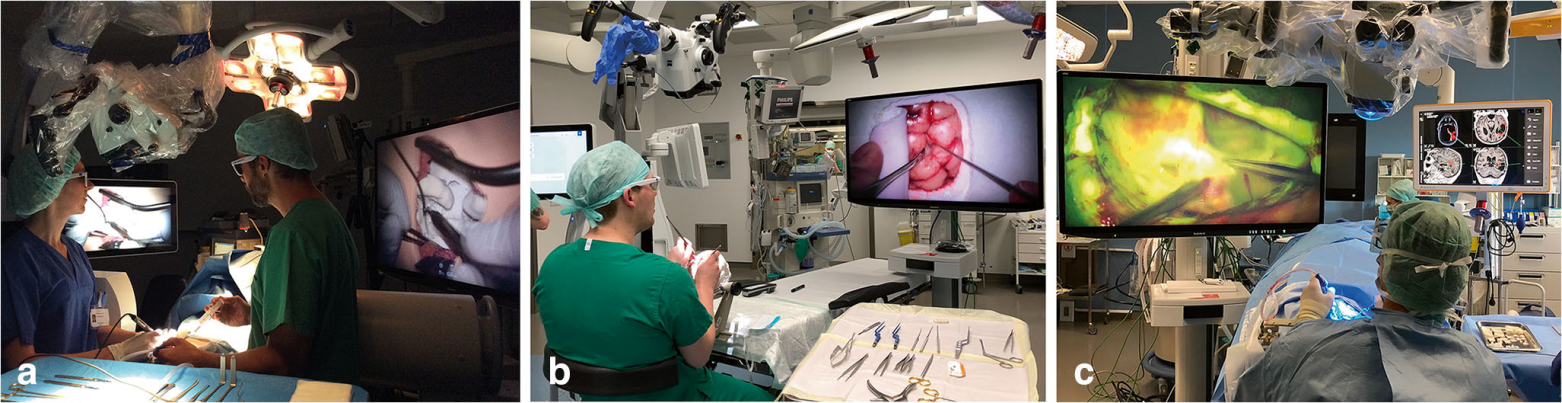
Preclinical setup: face-to-face scenario in spinal task with two 3D monitors (**a**); training scenario in cranial task (**b**); intraoperative setup: ›surgical cockpit‹ with 3D4k monitor and navigation screen, camera positioned perpendicular above surgical site (c)

The investigation addressed both quantitative and qualitative aspects of the device utilization in two phases (preclinical/clinical). In a two-day preclinical workshop in an actual OR environment, 12 neurosurgical professionals (6 residents PGY-2 to 5 and 6 attendings) performed predefined tasks on a skull/brain and spine phantom in a simulated team setting (see Fig. 1a–b). The tasks involved microsurgical vessel preparation and anastomosis (I) or tissue and nerve preparation (II) in a chicken wing/thigh model fixated in a skull simulator (Neuro Patient ›Graf‹, Phacon, Leipzig, Germany), as well as lumbar decompression (III) in a spine simulator (Lumbar Spine Patient ›Schumann‹, Phacon, Leipzig, Germany). Setup and task performance have been documented in each participant followed by a questionnaire with a 4-point Likert scale (1: excellent, 4: not acceptable) containing 30 items total (see supplementary material). The questionnaire results served as a basis for the definition of items in study phase 2. All items have been weighted equally.

After clinical introduction, the exoscope has been employed in a range of neurosurgical interventions (see Table 2, Fig. 1c). User experience has been rated on an adjusted 5-point Likert scale (1: excellent, 5: not acceptable) regarding setup and ergonomics, workflow, usability, and image quality. The randomized study phase consisted of a parallel group trial with 1:1 allocation ratio (equal numbers of prepared tickets for both groups); the same expert neurosurgeons (*n* = 4) performed either conventional or exoscopic procedures. A series of 20 subsequent uncomplicated supratentorial brain tumors has been randomly assigned to either monitor group (MG) or ocular group (OG) within 24 h before intervention. The study team enrolled and assigned patients; further inclusion criteria were no challenging microneurosurgical tasks involved, little expected blood loss, no to mild multimorbidity (ASA 1–2), age > 18 years. Switching to the other operating mode respectively has been optional at any time. The intraoperative documentation included microscope recording, setup photos, a structured observer assessment sheet containing pre-defined items plus a short interview allowing for additional user comments. Assessment parameters were surgical time, conversion of modality, ergonomics, surgical performance, setup specifications, workflow, and satisfaction with image quality. We used validated questionnaires from the System Usability Scale (SUS) [8] to assess different dimensions of usability and the Surgery Task Load Index (SURG-TLX) [30] to measure surgical workload. Ethical approval has been obtained from the local IRB (EA2/081/18), and written consent from both patients and surgeon participants was collected prior to intervention. The study has been registered with the German Clinical Trials Register (DRKS00016674). Results of the randomized clinical evaluation are reported according to the 2017 CONSORT NPT extension [7].

**Table 2:** Overview of cases in clinical phase (*n* = 29)]

Type	Group	Age	Sex	Pathology	Access	Short comment on exoscope utilization
Cranial (CE)	-	49	F	GBM left insular	Ext. pterional	Slowdowns, FCP conflicts, complex case
Spinal (CE)	-	42	F	Lumbar recessus stenosis	Lumbar	Good for in-focus tasks, control via handles faster
Cranial (CE)	-	38	M	GBM (rec) right temporal	Temporal	Time pressure, camera angulation too lateral
Cranial (CE)	-	54	F	GBM left perisylvian	Temporal	Suboptimal 3D quality, blurring, complex case
Cranial (CE)	-	68	F	Trigeminal nerve decompression	Retrosigmoidal	Improved ergonomics, keyhole-effect in depth
Cranial (CE)	-	24	M	CSF fistula	Frontal	Improved ergonomics
Cranial (CE)	-	52	M	aAST (rec) left frontal	Frontal	Camera angulation too lateral
Cranial (CE)	-	58	M	GBM left parietooccipital/opercular	Parietal	Camera angulation too lateral
Cranial (CE)	-	50	F	MNG right sphenoid wing	Pterional	Comfortable switch with macroscopic vision
Cranial (RCT)	OG	67	F	CM left precentral	Temporal	-
Cranial (RCT)	MG	70	F	MNG left parietal	Parietooccipital	Conversion for tumor-tissue contrast
Cranial (RCT)	MG	46	F	MNG left precentral	Frontal	Slowdowns, FCP conflicts
Cranial (RCT)	MG	78	M	GBM right parietal	Parietooccipital	Circumnavigation of resection cavity slow with FCP
Cranial (RCT)	OG	79	F	aODG (rec) right postcentral	Frontoparietal	-
Cranial (RCT)	MG	38	F	GBM (rec) right frontal	Frontoparietal	Fluoresceine: tissue contrast suboptimal due to brightness, but small vessels better detectable
Cranial (RCT)	OG	30	F	aAST (rec) left frontal	Frontoparietal	-
Cranial (RCT)	MG	43	M	aODG (rec) left frontal	Frontoparietal	No multivision info under fluoresceine
Cranial (RCT)	OG	59	F	GBM left suprasylvian	Temporal	-
Cranial (RCT)	MG	32	M	Met left occipital	Occipital	Handles control instead of FCP
Cranial (RCT)	MG	67	M	GBM (rec) right temporoparietal	Temporal	Camera angulation too lateral
Cranial (RCT)	OG	57	M	GBM left frontal	Frontal	-
Cranial (RCT)	OG	56	M	Met (rec) left parietal parafalcine	Parietal	-
Cranial (RCT)	MG	32	M	aODG (rec) left frontal	Frontoparietal	Very good visibility of target structures
Cranial (RCT)	OG	33	M	aAST left postcentral	Parietal	-
Cranial (RCT)	OG	79	M	Met right parietooccipital	Occipital	-
Cranial (RCT)	MG	29	M	MNG right precentral	Frontoparietal	Conversion for tumor-tissue contrast
Cranial (RCT)	OG	71	F	MNG right frontal	Frontoparietal	-
Cranial (RCT)	OG	75	M	MNG right postcentral	Parietal	-
Cranial (RCT)	MG	52	M	ODG right frontal	Pterional	Slowdowns; improved ergonomics

*aAST* anaplastic astrocytoma, *aODG* anaplastic oligodendroglioma, *CE* clinical exploration, *CM* cavernoma, *CSF* cerebrospinal fluid, *GBM* glioblastoma, *Met* metastasis, *MNG* meningioma, *MG* monitor group, *ODG* oligodendroglioma, *OG* ocular group, *RCT* randomized controlled trial, *rec* recurrence

### Metrics of surgical performance

3D stereoscopic vision [5] has been tested in all participants with a commercially available and validated stereopsis screening test (Frisby Pocket Stereo Test, Stereotest Ltd., Fulwood, UK, 2013). For assessment of surgical performance, we combined quantitative performance data (surgical time, EOR, complications, functional outcome) with semi-quantitative and qualitative performance data (surgical task completion, usability experience), using video analysis, scientific observation, and self-assessment. For investigation of potential impact on hand-eye coordination, particularly in the endoscopically untrained user [25], we determined a set of quantifiable indicators for surgical tool-tissue interaction [10, 12, 28], including hesitations, repetitions/corrections, slowdowns, monitor-to-site checks, and added surgical or task-completion time. For comparison during the study, we summed up these indicators in a cumulative hand-eye coordination score (CHECS). Observation and video analysis were performed by the study team. A blinded expert observer was consulted to assess and categorize the microscope recordings as ›ocular‹ or ›exoscope‹ mode, based on surgical tool handling and microscope command.

### Statistical analysis

All data was analyzed descriptively after completion of the study (SPSS Statistics version 25, 2017, IBM, Armonk/NY, USA). Significant differences between means were determined using a two-sample *t* test. We further used the Mann-Whitney *U* test for analysis of group differences in usability ratings including SUS and SURG-TLX results; Spearman’s coefficient has been employed for determining the association of setting alterations (e.g., camera angle) and performance (e.g., hand-eye coordination) during preclinical evaluation. Two-tailed probability values of *P* < .05 were considered statistically significant.

## Results

### Preclinical phase

#### Setup and ergonomics

Preferred positions of the external monitor were next to the OR table in direct sight axis to the surgeon with the exoscope positioned overhead. In face-to-face spinal setups the assistant surgeon (S2) has the same camera view as the operating surgeon (S1), which means for S2 a 180° rotated image making effective operative assistance impossible. Due to the limited working distance, exoscopic spine procedures are also affected by the surgeon’s body height (< 165 cm optimal) (see Fig. 1a). During exoscopic mode when standing, head posture was upright in 95% and upper body posture was upright in 98.3% of the time, compared with when sitting, head posture was upright in 87.1% and upper body posture was upright in 90.8% of the time (opposed to an average of 70% upright position in ocular mode for both postures when sitting).

#### Task performance

**Fig. 2:**
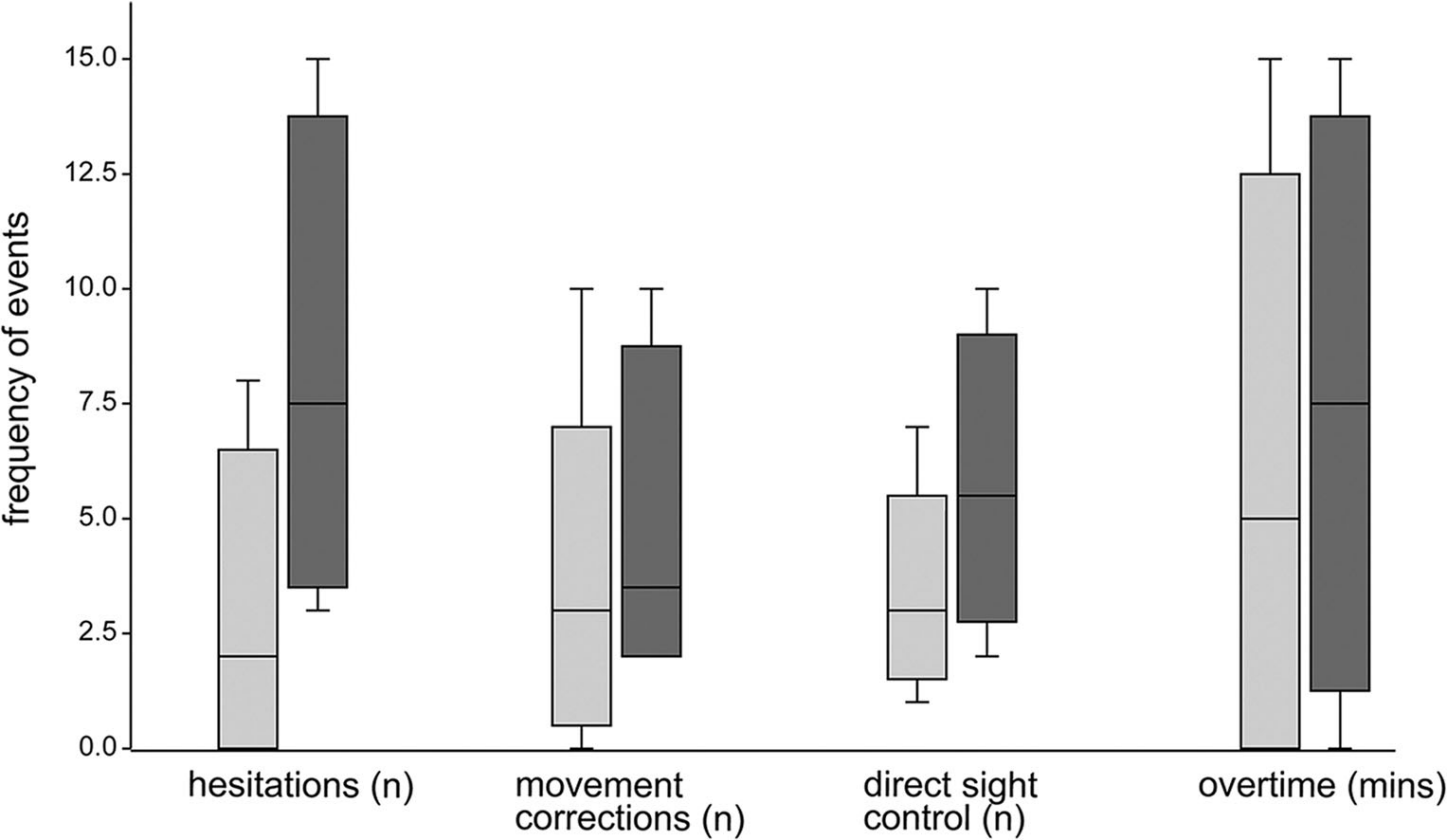
Synopsis of effects on hand-eye coordination in cranial task performance compared for frontoparietal (light gray) and pterional (dark gray) approaches (participants *n* = 9); plotted on the horizontal axis are numbers of hesitations, corrections, and direct sight control plus overtime needed for task completion in minutes

All participants were able to complete the tasks. Ten of 12 surgeons (83.3%) had a higher rate of movement hesitations and corrections in monitor-based performance. Monitor-to-site angles > 45° caused high discomfort. In monitor-to-site angles < 45°, we observed a linear increase of hesitations, corrections, and monitor-to-site checks (angle 0–10°: mean CHECS 14.6; angle > 10°: mean CHECS 20.7). Furthermore, we saw differences between more paramedian (frontoparietal) and more lateral (pterional) approaches (see Fig. 2) including a positive correlation between more lateral camera inclination and impact on hand-eye coordination (*r*_s_ = 0.756, *P* = .01).

#### Usability

Overall participant ratings across all 30 questionnaire items were good (median 2.0); only item 12 ›system monitor usability in S2‹ was ranked distinctly lower with median 4.0 (not acceptable) (see supplementary material, Table 3). Free text comments highlighted improved team communication and ergonomics on the one hand, and impaired hand-eye coordination and complicated hand-free camera control on the other hand (see supplementary material, Table 4).

#### Image quality

**Fig. 3:**
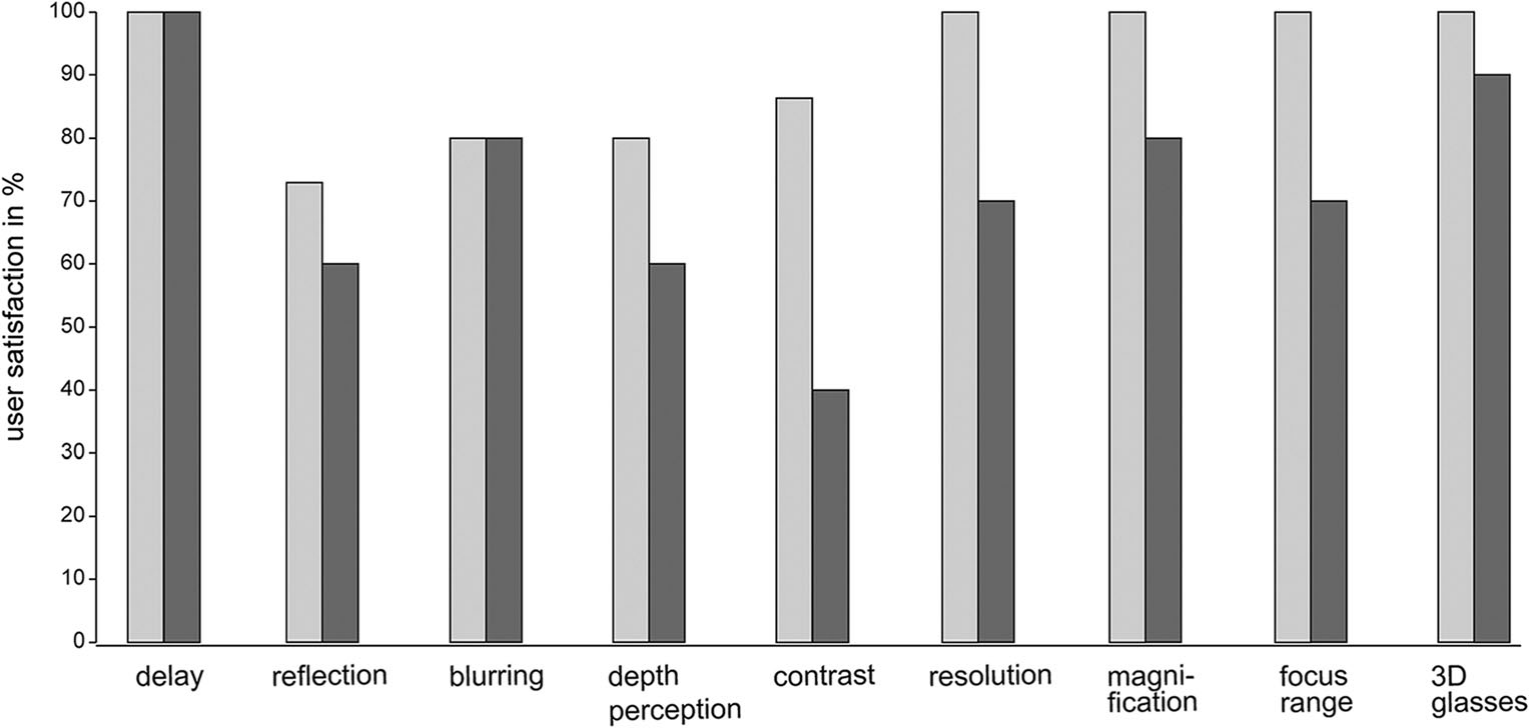
Dimensions of image quality satisfaction (%) present in digital visualization, compared between preclinical (light gray) and clinical (dark gray) settings

Image quality assessment focused on visual interferences (transmission delay 0%, monitor reflection 26.7%, image blurring 20%), satisfactory depth vision (80%), contrast (86.7%), and screen resolution (100%) (see Fig. 3). In 5 participants (41.7%), image quality has been rated inferior to ocular mode due to quality discrepancies between in-focus and off-focus areas.

### Clinical phase

All participants tested with the Frisby Pocket Test had a stereoacuity of ≤ 75 s arc, thus showing no indication of altered stereovision. Due to current limitations in spinal interventions, the focus of clinical case evaluation (CE) was on various types of cranial surgeries (see Table 2).

#### Clinical data

Tumor pathologies included 25% glioblastoma, 25% meningioma, 15% anaplastic oligodendroglioma, 15% metastasis, 10% anaplastic astrocytoma, 5% oligodendroglioma, and 5% cavernoma (mean patient age 55 ± 18 years, range 29–79; 60% male; 65% initial diagnosis; 55% left-sided lesions; 50% frontal, 30% parietal, 10% temporal, 10% occipital lesions; for legend of abbreviations and further information, see Table 2). Planned complete (CR) or gross total resection (GTR) could be achieved in 94% of cases, based on imaging results. Ten percent of cases (*n* = 2) had minor intraoperative complications (increased blood loss, subacute PICA infarction), which were however limited to the ocular group. Postoperative functional outcome was 80% idem, 10% impairment, 5% transient deficit, and 5% improvement.

Patient characteristics per group included a mean age of 61 years, 50% male, 40% eloquent location, 30% recurrence, 10% new permanent deficits, and 60% malignant/WHO grade III or higher tumors (OG) vs. a mean age of 49 years, 70% male, 10% eloquent location, 40% recurrence, 10% new permanent deficits, and 60% malignant/WHO grade III or higher tumors (MG). Fluorescence guidance has been used according to suspected pathology in 40% (OG) and 50% (MG) of cases respect**iv**ely.

#### Setup and ergonomics

**Fig. 4:**
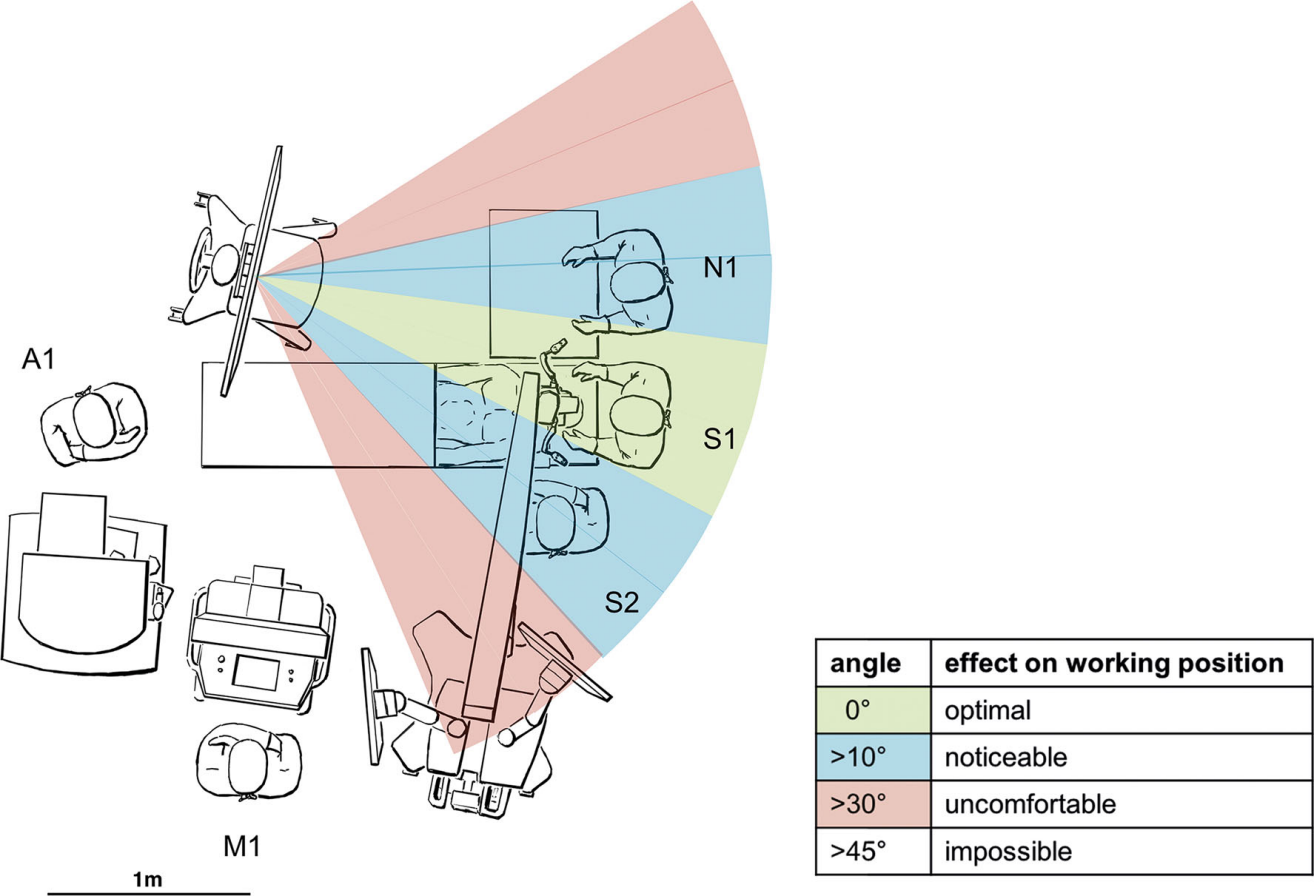
Intraoperative setup in cranial interventions with surgical assistant (S2), scrub nurse (N1), neuromonitorist (M1), and anesthesiologist (A1) showing effects of monitor-to-site angle on surgical performance

Setup integration has been achieved successfully in different neurosurgical ORs (see Fig. 1c). Mean monitor distance and angle were 180 ± 20 cm and 10 ± 10°. A right-sided position of the external monitor was preferable whenever a surgical assistant (S2) was present (see Fig. 4). The alignment of working and viewing direction (i.e., monitor-to-site angles < 10°) has been identified as a precondition (see Fig. 4). Overall surgical ergonomics when sitting improved in MG compared with OG (mean percentage of upright head posture during resection MG 72% vs. OG 52%, 95% CI: − 52.2–12.2; *P* = .21; mean percentage of upright upper body posture during resection MG 80% vs. OG 56%, 95% CI: − 44.6 to− 3.4; *P* = .03).

FCP integration turned out challenging in surgeries with two or more foot control panels in active use. Clinical user ratings showed a median score of 3.0 (acceptable) for all items with the following items rated difficult: ›off-focus tasks‹, ›S2 integration‹ and ›autofocus‹ (median 4.0). Highest-ranked items were ›ergonomics‹ and ›in-focus tasks‹ (median 2.0).

#### Workflow

There was no significant added surgical time in MG (mean overall surgical time: MG 220 ± 61 mins, 95% CI: 177–264, vs. OG 236 ± 67 mins, 95% CI: 188–284; *P* = .63; mean resection time MG 45 ± 23 mins, 95% CI: 28–61, vs. OG 34 ± 16 mins, 95% CI: 22–45; *P* = .28). The conversion rate in MG was 50% with case-dependent issues in lateral camera inclination and visual tissue differentiation. There was no conversion from OG to MG. Average monitor time in MG was 30 ± 20 mins (68% of MG mean resection time). Added preparation time was 10 mins on average.

#### Usability

**Fig. 5:**
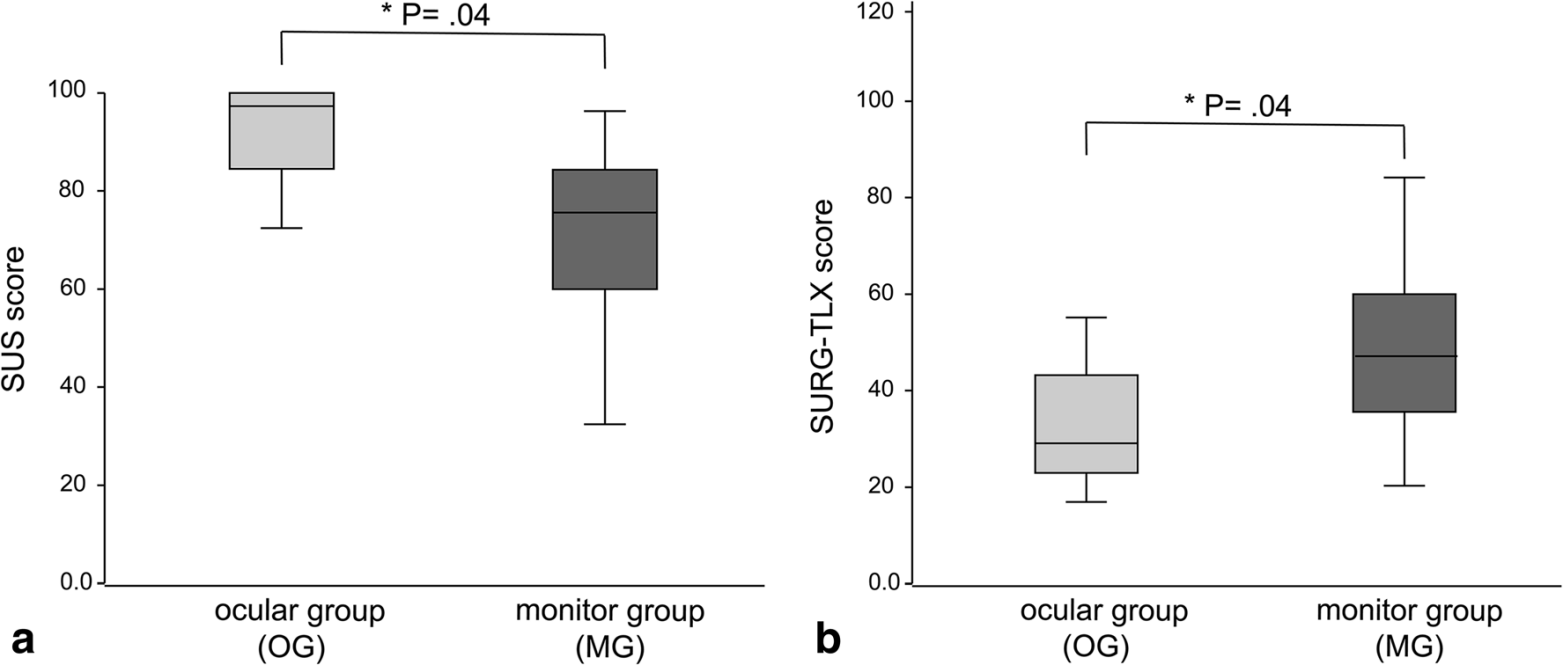
System Usability Scale (SUS) (**a**) and Surgery Task Load Index (SURG-TLX) (**b**) across all cases compared for both groups (OG, MG), showing an overall decreased mean usability and an increased mean workload in MG respectively with single users achieving comparable scores in both operating modes

An impact of exoscopic surgery on surgical hand-eye coordination has been observed in the majority of cases (70% hesitations, 80% corrections/repetitions, and 80% slowdowns) during the first 7.5 ± 2.5 mins of resection. The blinded expert rater was able to identify 68% of the case modes correctly, only being mistaken in the cases of the one expert user who did the most exoscopic surgeries in this study. FCP usability has been rated lower in the clinical phase (median preclinical 2.0 = good vs. clinical 3.5 = difficult). It was considered the most useful for small position refinements. SUS (score range 0–100, high score indicates high usability) showed significant group differences in items 1 ›readiness to use‹ (median MG 3.5 vs. OG 5.0; *P* < .001), 3 ›usability‹ (median MG 4.0 vs. OG 5.0; *P* < .001), 9 ›confidence‹ (median MG 4.0 vs. OG 5.0; *P* = .02) and overall score (mean MG 76.3 ± 22,5 vs. OG 93.5 ± 9,1, 95% CI: 0.53–33.97; *P* = .04) (see Fig. 5a). SURG-TLX (score range 6–120, high score indicates high surgical workload) showed significant group differences with increase of overall score (mean MG 47.2 ± 19.2 vs. OG 31.8 ± 12.3, 95% CI: − 30.51 to − 0.29; *P* = .04) and item 6 ›distraction‹ (median MG 4.5 vs. OG 3.0; *P* = .04) in MG (see Fig. 5b).

#### Image quality

MG satisfaction ratings in image quality were lower compared with ocular mode (screen resolution 70%, image contrast 40%, depth perception 60%, magnification 80%, focus range 70%, 3D glasses 90%; see Fig. 3). Main shortcomings stated were color accuracy, illumination, and image contrast when applied to tumor-tissue differentiation in detection of tumor borders.

## Discussion

The principle novel findings of our study comprise the overall clinical feasibility, safety, and ergonomic advantageousness of 3D4k hybrid exoscopic surgery in supratentorial brain tumors. We further analyzed shown restrictions in usability and visualization quality that limit the utilization of current generation exoscopes in more complex neurosurgical interventions.

### Clinical usability and eligible cases

In cranial interventions, our findings support a monitor distance of 180 cm and angle of 0–10° for the main surgeon with the monitor positioned on the scrub nurse side of the OR table.

By combining assessments of usability and task load, we could identify areas where further action (training, improved control devices) is needed in order to achieve user satisfaction (technological confidence) and reduce added contextual burden (distraction) for the surgeon. The critically limiting factor in device usability was the FCP controlling exoscope robotics. The full potential of exoscopic surgery will only be exploited once intuitive hand-free controls of the camera position are implemented. All participants agreed that with the greater field of view in exoscope mode, effects of distraction are more relevant than in ocular mode. This correlates with the significantly increased SURG-TLX score in item 6 ›distraction‹. In more remote positions of the external monitor, the effect of distraction increases.

Due to an interdependence of increasingly lateral camera angles and restricted visibility of structures in the depth of the resection cavity, frontoparietal and pterional approaches were better suited for exoscopic surgery than retrosigmoidal or suboccipital approaches. Supratentorial brain tumor cases that do not require a small surgical corridor are most suited for exoscopic surgery with a hybrid visualization system. Our use cases equal a level II complexity [11] with the option to increase surgical complexity with a long-term learning curve. Due to current limitations (S2 integration, S1 body height), spinal interventions are not suitable for exoscopic surgery with this system.

### Impact on performance

Most exoscope studies report improved ergonomics for the operating surgeon (see Table 1). However, the surgeon’s body posture is determined by the patient’s position, surgical access, and viewing angle to the surgical site. Still, we could show that, in direct comparison with the ocular mode, the exoscope is in many cases ergonomically favorable.

Hand-eye coordination is clearly affected in exoscope mode. During familiarization with the technology, similar issues could be observed as in 2D exoscopes and endoscopes: many surgeons used initially both hands in order to understand depth and range of their instrument handling, guiding one instrument with another. The adjustment time decreases with experience. Earlier reports [20] stated an adaptation period of approx. 60 minutes for hand-eye coordination in monitor-based 2D visualization—less, if the surgeon was trained in neuroendoscopy. Acclimatization time needed for optimal dexterity was further related to the depth of field.

### Quality of visualization

Image quality was rated more critically during the clinical study phase (see Fig. 3) when the requirements of image quality became case-specific. In exoscope mode, small focus ranges in the depth of a resection cavity limit in-focus working noticeably compared with the optic mode where physiologic eye accommodation can actually compensate for off-focus actions. Thus, the actual (in-focus) working area in deep-seated structures turns out smaller. The field of view is cut to fit the rectangular monitor, requiring more frequent camera position adjustments during surgery. Regarding tissue differentiation in brain tumor interventions, there are different positions in the literature [23] stating better, equal, or worse quality compared with optical microsurgery. However, image contrast including color accuracy was rated the lowest in this study; beside detection of tumor-tissue borders, fast visual identification of small bleeding vessels can be challenging. The utilization of a hybrid visualization system like the one investigated in this study can help adjust workflows and gain experience in more complex microneurosurgical tasks before switching to a standalone exoscope. Until completion of this paper, one other study on the same system has been published [4] presenting results of a comparative preclinical assessment of microvascular anastomosis. The authors conclude that the 3D4k exoscope allows for neurovascular task completion; however, in their evaluation, size and width of the field of view as well as sharpness and resolution at high magnification were still superior in ocular mode. We claim that established minimum visual requirements such as illumination, magnification, depth vision, and detail/overview are inadequate for high-quality exoscopic microneurosurgery. Rather, all image settings including brightness, color filters, contrast, resolution, and sharpness should be assessed and further investigated in detail, as they define the visual expectation in the trained expert neurosurgeon.

### Limitations of this study

This study has been designed to investigate the hybrid exoscope under clinical routine conditions in the neurosurgical OR. As a consequence of clinical feasibility and intraoperative workflow, we did not employ any advanced quantitative measuring systems for assessment of technical performance, such as eye and motion tracking devices. Higher numbers of cases and participating surgeons are required for further investigation of eligible procedures in exoscopic microneurosurgery. We would also like to note that microscope visualization and expert command of the technology will be even more critical in complex surgical cases. During the course of this study, an updated system with optimized image quality parameters was released (KINEVO 900 version 1.5) that could not be taken into consideration.

## Conclusions

3D4k exoscopic surgery is feasible in clinical routine with clearly defined limitations. Depending on surgical access and case, neurosurgical routine tasks can be performed unrestrictedly. Major advantages of the exoscope are improved ergonomics, an unblocked primary surgical field of vision and a shared 3D view for the complete surgical team offering increased educational value. Major limitations concern interventions through small surgical corridors and the high frequency of camera adjustments in monitor mode. Further disadvantages include a temporally impaired hand-eye coordination, imperfect hand-free control, increased spatial requirements in the OR, and limitations in image quality (brightness, contrast, color accuracy). In our case series, we observed a conversion rate of 50% from exoscope to microscope. Based on our results, we cannot recommend abandoning the established standard at this point. In future exoscopic neurosurgery, improvements in monitor, camera, and image filter technology are indispensable for routine application.

## Electronic supplementary material

ESM 1 (DOCX 22 kb)
