# Supplementary material for: Monitor-based exoscopic 3D4k neurosurgical interventions: a two-phase prospective-randomized clinical evaluation of a novel hybrid device
Source: Acta Neurochir (Wien). 2020 May 19;162(12):2949–61. doi: 10.1007/s00701-020-04361-2 (PMC7593287; doi:10.1007/s00701-020-04361-2)
Supplement: Supplementary file 1 — (DOCX 22 kb) [file 701_2020_4361_MOESM1_ESM.docx]

Supplementary Material:

User Questionnaire For Evaluation of Hybrid Exoscope Scenario

A During Surgery

1. How well does the position of the microscope allow the main surgeon free working space to handle surgical instruments and perform surgery in good view of the monitor?
   □ excellent □ good □ acceptable □ not acceptable
2. How reliably can the main surgeon have eye contact with the entire surgical team?
   □ excellent □ good □ acceptable □ not acceptable
3. How well does the position of the microscope provide the surgical assistant free working space and adequate visualization to assist surgery?
   □ excellent □ good □ acceptable □ not acceptable
4. How well does the position of the microscope provide the sterile nurse free working space and adequate visualization to assist surgery?
   □ excellent □ good □ acceptable □ not acceptable
5. How safely can the range and pace of the robotic microscope arm be safely adjusted in and around the surgical field by the main surgeon?

□ excellent □ good □ acceptable □ not acceptable

1. How well does the digital approach allow for additional freedom for the main surgeon including utilizing previously restricted microscope orientations?

□ excellent □ good □ acceptable □ not acceptable

1. How well could the main surgeonbenefit from utilization of autofocus functionality?

□ excellent □ good □ acceptable □ not acceptable

1. How well does the magnification functionality allow for a precise and comfortable assessment of the surgical field by the main surgeon?

□ excellent □ good □ acceptable □ not acceptable

1. How well does the digital visualization provide sharp tissue-tissue/tissue-pathology contrast to the main surgeon?

□ excellent □ good □ acceptable □ not acceptable

1. How acceptable is the frequency of alternate viewing directions (between site and monitor or additional screens) by the main surgeon when using the digital monitor image?

□ excellent □ good □ acceptable □ not acceptable

B After Surgery

1. How well can the surgical assistant share the monitor image with the main surgeon to assist the surgery?

□ excellent □ good □ acceptable □ not acceptable

1. How well can the surgical assistant utilize the system monitor at the microscope stand to assist surgery?

□ excellent □ good □ acceptable □ not acceptable

1. How reliably does the OR setup provide suitable position and space for the anesthesiologist?

□ excellent □ good □ acceptable □ not acceptable

1. How easily can the microscope and video monitor be integrated into the standard OR setup?

□ excellent □ good □ acceptable □ not acceptable

1. How effortlessly can the microscope and video monitor be positioned according to patient positioning?

□ excellent □ good □ acceptable □ not acceptable

1. How well can the sterile working zones of main surgeon, assistant surgeon and sterile nurse be integrated with microscope and monitor positioning?

□ excellent □ good □ acceptable □ not acceptable

1. How suitable are the estimated technical and workflow team training requirements regarding microscope and monitor for surgical routine?

□ excellent □ good □ acceptable □ not acceptable

1. How well does the position of the microscope allow the main surgeon to adjust the microscope to any needed position or orientation to observe the surgical site?
   □ excellent □ good □ acceptable □ not acceptable
2. How precisely and efficiently can the main surgeon complete the surgical task?
   □ excellent □ good □ acceptable □ not acceptable
3. How realiably does the digital visualization of the monitor image allow for precise hand-eye coordination of the main surgeon?
   □ excellent □ good □ acceptable □ not acceptable
4. How realistic is the 3D impression provided by the digital visualization for the main surgeon?
   □ excellent □ good □ acceptable □ not acceptable
5. How comfortable is the ergonomic position of the main surgeon and how reliably does it allow for working at high concentration on the surgical procedure?
   □ excellent □ good □ acceptable □ not acceptable
6. How reliably does the digital visualization provide a real-time iamge without noticeable latency for the main surgeon?
   □ excellent □ good □ acceptable □ not acceptable
7. How well does the digital visualization allow the main surgeon to perform surgical tasks without noticeable fatigue or other negative impact, such as e.g. nausea?

□ excellent □ good □ acceptable □ not acceptable

1. How well can the main surgeon concentrate on the surgical task without distraction or disturbance from the OR setting and activities of the surgical team?

□ excellent □ good □ acceptable □ not acceptable

1. How good is the experience of the main surgeon regarding movement hesitations and/or corrections when using the digital monitor image?

□ excellent □ good □ acceptable □ not acceptable

1. How good is the experience of the main surgeon regarding restrictions in range and speed of instrument handling when using the digital monitor image?

□ excellent □ good □ acceptable □ not acceptable

1. How precisely and effortlessly can the main surgeon utilize the foot control panel to adjust the microscopic setup, including positioning and focusing?
   □ excellent □ good □ acceptable □ not acceptable
2. How would you as the main surgeon rate the necessity to visually control your performance with the naked eye when using the digital monitor image?

□ excellent □ good □ acceptable □ not acceptable

1. How much can using Hybrid Digital Visualization be recommended for this surgical approach/test scenario?

□ excellent □ good □ acceptable □ not acceptable

Supplementary Material

Table 3: User Questionnaire For Evaluation of Hybrid Exoscope Scenario: Overview of Results

| Item No. | Short Description | Median Rating Results |
| --- | --- | --- |
| 1 | working space S1 | 1 |
| 2 | eye contact | 1 |
| 3 | working space S2 | 2 |
| 4 | working space N1 | 1 |
| 5 | robotic safety | 2 |
| 6 | microscope orientations S1 | 1,5 |
| 7 | autofocus S1 | 2 |
| 8 | magnification S1 | 1,5 |
| 9 | image contrast S1 | 1,5 |
| 10 | viewing directions S1 | 1,5 |
| 11 | use of 55” monitor S2 | 2 |
| 12 | use of small monitor S2 | 4 |
| 13 | working space A1 | 1,5 |
| 14 | integration into OR setup | 2 |
| 15 | patient position | 2 |
| 16 | sterility | 2 |
| 17 | OR workflow | 2 |
| 18 | microscope adjustments S1 | 1 |
| 19 | task completion S1 | 1 |
| 20 | hand-eye coordination S1 | 1 |
| 21 | 3D image S1 | 2 |
| 22 | ergonomics S1 | 1 |
| 23 | transmission delay S1 | 1 |
| 24 | fatigue S1 | 1 |
| 25 | distraction S1 | 2 |
| 26 | hesitations/corrections S1 | 2 |
| 27 | instrumentation S1 | 2 |
| 28 | FCP S1 | 2 |
| 29 | visual control S1 | 1,5 |
| 30 | overall satisfaction S1 | 1,5 |

abbreviations: A1 = anesthesiologist, FCP = foot control panel, N1 = sterile nurse, OR = operating room, S1 = main surgeon, S2 = assistant surgeon

Supplementary Material

Table 4: User Questionnaire For Evaluation of Hybrid Exoscope Scenario: Free Text Comments

| Item No. | advantages of hybrid exoscope | disadvantages of hybrid exoscope |
| --- | --- | --- |
| 1 | - | - |
| 2 | - improved team communication | - |
| 3 | - | - 90° position and view are problematic - monitor view restricted by obstacles - due to discrepancy of working and viewing angle - increased impact on hand-eye coordination - face-to-face setups currently not supported in 3D |
| 4 | - | - |
| 5 | - | - frequent collisions with head position |
| 6 | - improved ergonomics - in general comparable to ocular-based surgery | - brainstem approaches and other deep-seated lesions potentially difficult - only if using long instruments |
| 7 | - to date, only infrequently used | - |
| 8 | - | - loss of sharpness in high magnification |
| 9 | - | - ocular vision is superior |
| 10 | - parallel use of ocular and monitor image if required or convenient | - |
| 11 | - | - assistant has to be placed opposite to monitor - very limited view |
| 12 | - | - |
| 13 | - | - |
| 14 | - more variable settings | - monitor placement should be more flexible - ceiling-mounted solution preferable |
| 15 | - more variable angulations |  |
| 16 |  | - depending on distance of camera to site - risk of head collision |
| 17 | - | - |
| 18 | - adjustments can be performed more smoothly | - |
| 19 | - instrument handling facilitated by using foot control panel for camera positioning - broader area of view in monitor-based surgery | - except for detailed microneurosurgical tasks - depending on access route |
| 20 | - | - manipulating suture easier when using ocular |
| 21 | - | - restricted depth perception - field of view smaller than in ocular-based surgery - in small structures, ocular vision superior - working off-focus more cumbersome than in ocular-based surgery |
| 22 | - | - |
| 23 | - | - |
| 24 | - (comparable to ocular-based surgery) | - |
| 25 | - monitor size sufficient | - distraction caused by team members - (monitor position should be close to main surgeon) |
| 26 | - | - initial adjustment/training period required |
| 27 | - | - alternate views between monitor and surgical site |
| 28 | - easy to learn - hand-free control of visualization facilitates surgery - good grip and stability | - shoeless interaction recommended - too many buttons - too unprecise - handles preferable - additional training/learning curve required - potential conflicts with additional foot panels - waterproof cover required |
| 29 | - | - |
| 30 | - hybrid tool - suitable for routine cases - improved ergonomics - shared view for whole team | - restricted space in OR for circulating nurse |
